# Long-term survival of patients after ipilimumab and hypofractionated brain radiotherapy for brain metastases of malignant melanoma: sequence matters

**DOI:** 10.1007/s00066-018-1356-5

**Published:** 2018-10-08

**Authors:** Heinz Schmidberger, Matthias Rapp, Anne Ebersberger, Silla Hey-Koch, Carmen Loquai, Stephan Grabbe, Arnulf Mayer

**Affiliations:** 1 Department of Radiation Oncology, University Medical Center of the Johannes Gutenberg University, Langenbeckstr. 1, 55131 Mainz, Germany; 2 Department of Dermatology, University Medical Center of the Johannes Gutenberg University, Mainz, Germany

**Keywords:** Immunotherapy, Whole brain radiotherapy, Stereotactic radiotherapy, Immunogenic cell death, Multvariate analysis, Immuntherapie, Ganzhirnbestrahlung, Stereotaktische Radiotherapie, Immunogener Zelltod, Multivariate Analyse

## Abstract

**Purpose:**

Since the introduction of ipilimumab (IPI) for the treatment of patients with metastatic malignant melanoma, we have observed remarkable responses after hypofractionated whole brain irradiation (WBRT) or stereotactic radiotherapy (STX) for brain metastases of malignant melanoma. We sought to investigate the impact of the sequence of these treatment modalities.

**Methods:**

We retrospectively evaluated the survival of melanoma patients with brain metastases who were treated with WBRT or STX and received IPI in close temporal relation between October 2010 and March 2015. Follow-up was obtained until November 2016. A total of 27 patients with advanced melanoma and brain metastases who were treated with WBRT before 2010, and who had not received IPI, served as historical controls.

**Results:**

We identified a total of 41 patients of whom 15 were treated with STX, 7 with a combination of STX and WBRT and 19 with WBRT alone. All patients received at least 2 doses of IPI. The median time interval between radiotherapy and IPI was 2 months. Patients treated with IPI after radiotherapy had a censored median survival of 11 months, compared with 3 months for the patients who received IPI prior to radiotherapy. Patients who received IPI before radiotherapy showed a similar survival as historical controls, who had not received IPI. We observed long-term survivors after radiotherapy of brain metastases followed by IPI.

**Conclusions:**

These data suggest that the sequence of RT and immune checkpoint inhibition with IPI may be crucial for the success of combined modality treatment of melanoma brain metastases.

## Zusammenfassung

**Zielsetzung:**

Seit der Einführung von Ipilimumab (IPI) zur Behandlung von Patienten mit metastasierendem malignem Melanom haben wir Verläufe mit bemerkenswertem Ansprechen von Hirnmetastasen nach hypofraktionierter Ganzhirnbestrahlung (WBRT) oder stereotaktischer Radiotherapie (STX) beobachtet. Hier haben wir untersucht, ob bzw. wie sich die zeitliche Abfolge des Einsatzes dieser Behandlungsmodalitäten auswirkt.

**Methoden:**

Wir haben retrospektiv das Überleben von Melanompatienten mit Hirnmetastasen untersucht, die zwischen Oktober 2010 und März 2015 mit WBRT oder STX behandelt worden waren und in engem zeitlichen Zusammenhang damit IPI erhalten hatten. Nachbeobachtungsdaten wurden bis November 2016 erhoben. Siebenundzwanzig Patienten mit fortgeschrittenem Melanom und Hirnmetastasen, die vor 2010 mit WBRT behandelt worden waren und kein IPI erhalten hatten, dienten als historische Kontrollen.

**Ergebnisse:**

Wir haben insgesamt 41 Patienten identifiziert, von denen 15 mit STX, 7 mit einer Kombination von STX und WBRT und 19 mit alleiniger WBRT behandelt worden waren. Alle Patienten hatten mindestens 2 Dosen IPI erhalten. Das mediane Zeitintervall zwischen Strahlentherapie und IPI betrug 2 Monate. Patienten, die nach einer Strahlentherapie mit IPI behandelt wurden, hatten eine mediane Überlebenszeit von 11 Monaten, während diese für Patienten, die vor der Strahlentherapie IPI erhielten, nur 3 Monate betrug. Patienten, die IPI vor der Strahlentherapie erhalten hatten, zeigten ein ähnliches Überleben wie historische Kontrollen, die kein IPI erhalten hatten. Wir beobachteten Langzeitüberlebende nach Strahlentherapie von Hirnmetastasen gefolgt von IPI.

**Schlussfolgerungen:**

Diese Daten legen nahe, dass die zeitliche Abfolge der Applikation von Radiotherapie und Immun-Checkpoint-Hemmung mit IPI ein entscheidender Faktor für den Erfolg dieser Kombinationstherapie bei Hirnmetastasen eines malignen Melanoms sein könnte.

## Introduction

Over one-third of patients with malignant melanoma eventually develop clinically apparent brain metastases during the course of their disease [1]. Patients with brain metastases have a significantly worse progression-free survival (PFS), and overall survival (OS) compared to those with metastases confined to other organs [2]. In most cases, brain metastases are the limiting factor for OS, as the majority of patients will die of neurological complications [3]. Until recently, whole brain radiation therapy (WBRT) in combination with stereotactic radiotherapy (STX) or neurosurgery represented the most effective treatment for these patients. However, median survival was between 3 and 9 months [4], and long-term survivors had never been seen [3]. Most prospective studies which evaluated ipilimumab (IPI) in patients with malignant melanoma have excluded patients with brain metastases. Therefore, prospective studies on the efficacy of IPI in patients with brain metastases are lacking. Only retrospective data and case reports have been published so far.

**Fig. 1:**
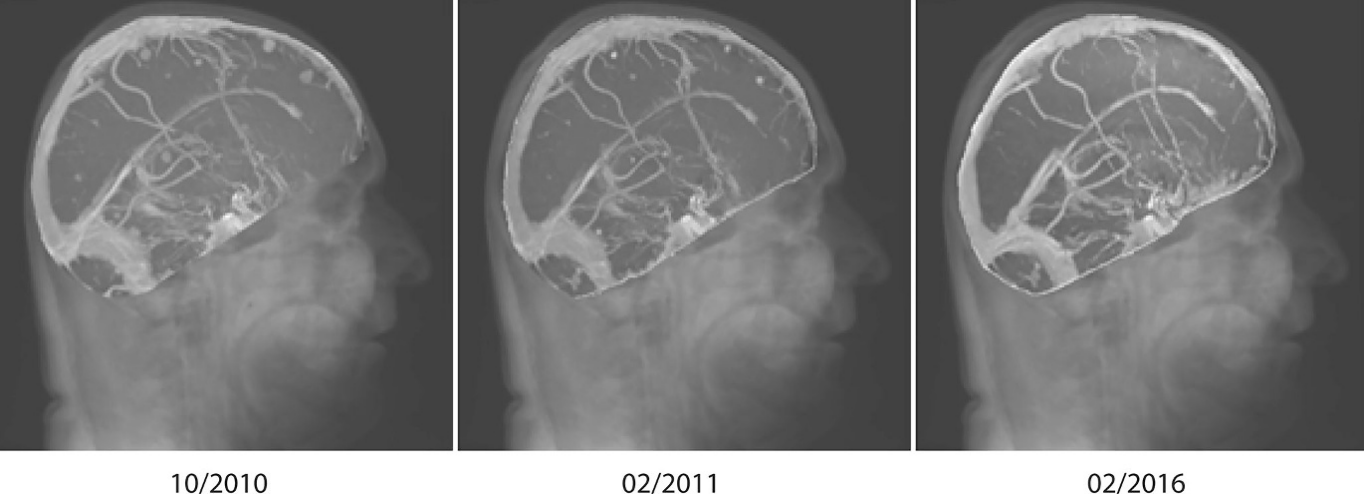
Maximum intensity projection (MIP) view of T1-weighted magnetic resonance images (MRI) immediately following whole brain irradiation (WBRT; 10/2010), 1 month after completion of 4 cycles of ipilimumab (IPI; 02/2011) and 5 years later (02/2016), showing complete remission (RECIST 1.1) of brain lesions

In 2010, IPI became available for compassionate use in patients with stage IV melanoma. One of the patients who received IPI in our center had just previously undergone whole brain radiotherapy for inoperable multiple brain metastases. Surprisingly, this patient achieved a complete remission and is alive and disease free more than 5 years after the IPI treatment (Fig. 1). This observation and the ongoing discussion about the relevance of the sequence of the application of radiotherapy and immunotherapy [5–7] prompted us to analyze the clinical course of all patients who had received IPI and radiotherapy in close temporal association, to identify possible determinants for the fruitful interaction of both treatment modalities. Patients who had received IPI and radiation to brain metastases were compared to historical controls, who had received radiotherapy alone, before the availability of IPI.

## Patients and methods

We retrospectively examined two cohorts of patients with brain metastasis of melanoma. The first group consisted of all patients who had been treated at our institution with a combination of radiotherapy to the brain and IPI (*n* = 41) between October 2010 and March 2015. All of these patients underwent hypofractionated whole brain radiotherapy (WBRT, *n* = 19), stereotactic radiotherapy (STX, *n* = 15) or a combination of both techniques (*n* = 7) and received IPI, predominantly in a narrow time interval before or after irradiation. All patients received at least two applications and a maximum of four applications of IPI. A total of 39 patients received a dose of 3 mg/kg, while 2 patients received 10 mg/kg intravenously, applied every 3 weeks in each case.

Historical patients from our clinic (*n* = 27) served as a control group. The latter had received whole brain irradiation during the years 2006–2009, either to a total dose of 30 Gy in single doses of 5 × 3.0 Gy per week or up to a total dose of 25 Gy in two single daily fractions of 2.5 Gy, also 5 × per week. All of the patients from that period were included. These patients received neither IPI nor any other form of immune checkpoint inhibition. In both groups, participants could have had any number and any kind of previous conventional treatments such as dacarbazine, paclitaxel/carboplatin, interferon-α, or vemurafenib (see Table 1). This study has been approved by the ethics committee of the Landesärztekammer Rheinland-Pfalz (No. 837.281.17 (11115)).

**Table 1:** Patient data according to the sequence of ipilimumab (IPI) and irradiation

		IPI before irradiation (*n* = 20)	IPI after irradiation (*n* = 21)	Difference between groups (*p*-values)
*Age (years)*				
Median (range)		62.5	52	0.28
Mean		61	53	0.05
*Sex*				
Male		70% (14)	67% (14)	1.00
Female		30% (6)	33% (7)	
*RPA Classification*				
3		40% (8)	33% (7)	0.66
2		60% (12)	67% (14)	
1		0% (0)	0% (0)	
*Mode of Radiotherapy*				
STX alone		45% (9)	29% (6)	0.34
STX + WBRT		15% (3)	19% (4)	1.00
WBRT alone		40% (8)	52% (11)	0.54
*Surgical resection*		15% (3)	33% (7)	0.24
*No. of cycles of IPI*				
Median (range)		4	4	0.97
Mean		3.5	3.7	0.72
*Time interval between irradiation and IPI (months, relative to begin of irradiation)*				
Median		−3 (−28; 0)	+1 (0; 21)	0.65
Mean		6.6	4	0.24
*Further systemic therapy* ^*a*^				
Inhibition of MAPK signaling	BRAF inhibitor	25% (5)	48% (10)	0.20
	MEK Inhibitor	15% (3)	15% (3)	1.00
Anti PD-1/PD-L1		20% (4)	15% (3)	0.70
Conventional therapy^b^		80% (16)	81% (17)	1.00

*RPA* recursive partitioning analysis, *MAPK* mitogen-activated protein kinase, *BRAF* v-raf murine sarcoma viral oncogene homolog B, *MEK* MAPK/ERK kinase, *PD-1* programmed cell death protein 1, *PD-L1* programmed cell death 1 ligand 1

^a^Some patients received multiple treatments

^b^Interleukin 2, Interferon α, polychemotherapy regimens (dacarbazine, temozolomide, paclitaxel, carboplatin)

As time-to-event endpoints, this retrospective cohort study used overall survival (OS) and cerebral progression-free survival (CPFS), which were estimated with the Kaplan–Meier product limit method and log-rank statistics. CPFS data were not available for the historical control patients. Survival times were calculated starting with the first day of brain radiotherapy. A multivariate analysis was done using the Cox Proportional Hazards model and the “enter” method (see results section for additional details). Fisher’s exact test was used to calculate possible differences in the distribution of categorical variables between patient groups. All analyses were carried out in SPSS Version 23.0 (IBM, Armonk, NY, USA). The effect of corticosteroids on outcome measures was not considered since the number of patients is too low to analyze this possible confounding factor. In our cohort, most patients did receive corticosteroids at least at the beginning of brain irradiation. Furthermore, we used the Radiation Therapy Oncology Group (RTOG) recursive partitioning analysis as a prognostic score, as described by Gaspar et al. [8]. According to these authors, the following definitions apply. Class 1: Karnofsky Performance Status (KPS) ≥70, <65 years of age with controlled primary and no extracranial metastases; Class 3: KPS <70; Class 2: all others.

## Results

**Fig. 2:**
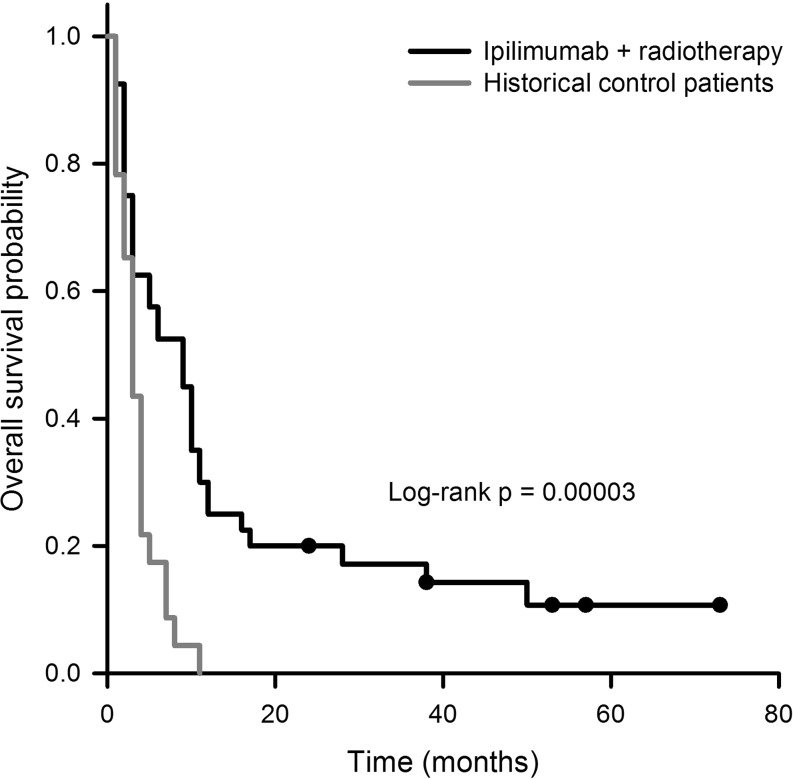
Overall survival probability of patients treated with a combination of ipilimumab (IPI) and radiotherapy (*n* = 41) compared with historical controls (*n* = 27)

The median overall survival (OS) in the cohort treated with radiotherapy plus IPI was 9.0 months, the median cerebral progression-free survival (CPFS) was 3.0 months. After 36 months, 4 patients of this group were still alive, and 3 patients have been living longer than 48 months. The median OS of the historical controls was 3.0 months. All patients of this cohort have died, with the maximum survival time being 11.0 months and the median survival time 3.0 months. The difference in overall survival between the experimental group (radiotherapy plus IPI) and the historical controls was statistically highly significant (*p* = 0.00003, Fig. 2).

**Fig. 3:**
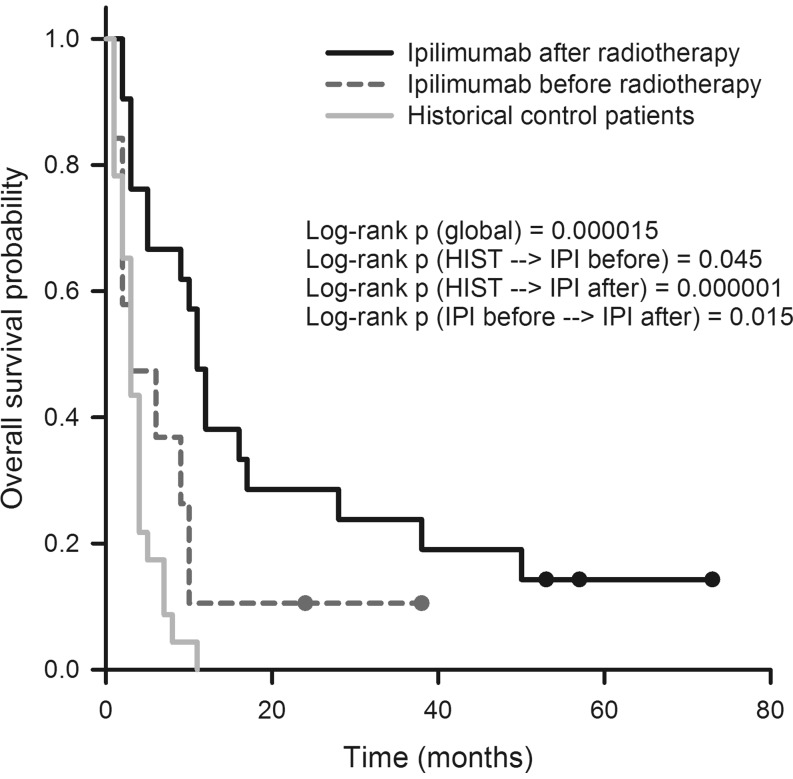
Overall survival probability of patients who had received radiotherapy before ipilimumab (IPI) compared with the inverse sequence and historical controls. See text for statistical differences between groups. *HIST* historical control patients

**Fig. 4:**
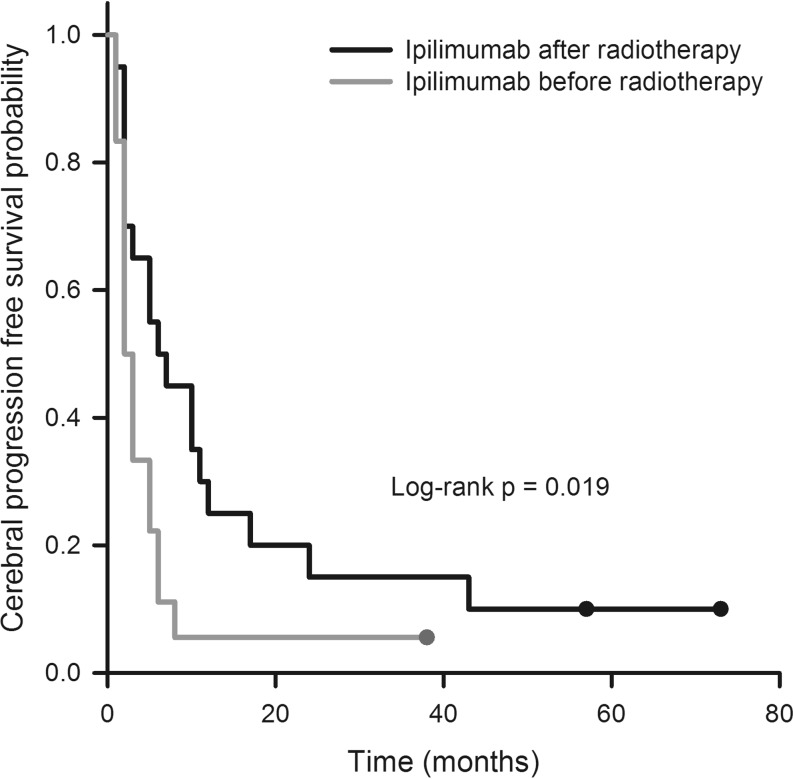
Cerebral progression-free survival probability of patients who had received radiotherapy before ipilimumab (IPI) compared with the inverse sequence

Next, we analyzed the survival data after further dichotomizing the experimental cohort in patients who received IPI before or after irradiation. Interestingly, these two groups were virtually of the same size (IPI before radiotherapy, *n* = 20, IPI after radiotherapy, *n* = 21). With a median survival time of 11.0 months, patients who received IPI after irradiation had the best OS as compared not only with the historical controls (3.0 months, *p* = 0.000001) but also with the patients who had received IPI before irradiation (3.0 months, *p* = 0.015). The difference between the two previously mentioned groups (IPI before radiotherapy vs. historical controls) was only marginally significant (*p* = 0.045, Fig. 3). Regarding CPFS, patients who had received IPI after radiotherapy again had a significantly more favorable outcome than those who had been treated with IPI before radiotherapy (6.0 vs. 2.0 months, *p* = 0.019). Owing to the lack of CPFS data for the historical controls, no comparison with this patient cohort was possible (Fig. 4).

These findings resulted in the question, as to whether an unequal distribution of patient- or tumor-associated factors in the two experimental groups (IPI before or after radiotherapy) could explain these striking differences with regard to prognosis. Of the factors age, gender, recursive partitioning analysis (RPA) class, radiotherapy modality, surgical tumor resection, and number of cycles of IPI, only age and previous surgery were found to show a marginally unequal distribution (Table 1). Patients who received IPI after radiotherapy were on average 8 years younger. The difference between the two groups using the t‑test with a *p*-value of 0.05 just missed the level of statistical significance. Patients who had undergone surgery were also more frequently present in the group treated with IPI after radiotherapy, but again, this was not statistically significant (29% vs. 10%, *p* = 0.24). Finally, the proportion of patients who had previously received BRAF inhibitors was almost twice as high in the “favorable” group of patients who had received IPI after radiotherapy. Again, however, this difference failed to reach formal statistical significance.

Further univariate survival analyses revealed a better survival for patients aged ≤59 years compared to those 60 years of age or older (OS: 10 months vs. 3 months, *p* = 0.025; CPFS: 5 months vs. 2 months, *p* = 0.017), for patients assigned to the lower RPA class (OS: 10 months vs. 3 months, *p* = 0.003, CPFS: 5 months vs. 2 months, *p* = 0.043) and for the patients who had undergone surgery (OS: 16 months vs. 5 months, *p* = 0.049, CPFS: 10 months vs. 2 months, *p* = n. s.). Conversely, no statistically significant differences were found between the genders and groups of patients treated with different techniques of radiotherapy (WBRT alone, STX alone, or a combination of STX and WBRT).

Since they were found to have a significant impact on survival in the univariate analysis, the variables sequence (IPI before vs. after radiotherapy), age (dichotomized using the median as the cut-off), surgery and RPA class were subjected to a forced inclusion into a multivariate Cox Proportional Hazards model (see methods section). For the OS, only the higher RPA class (HR 3.3, *p* = 0.003) and the administration of IPI before radiotherapy were found as independent factors of a poorer prognosis (HR 2.7, *p* = 0.01). Regarding CPFS, the administration of IPI before radiotherapy alone remained the only independently significant factor of a worse prognosis (HR 2.1, *p* = 0.045, see Table 2).

**Table 2:** Results of the uni- and multivariate survival analyses

**Univariate analysis**					
		**Overall survival (months)**	***p*** **-value**	**Cerebral progression-free survival (months)**	***p*** **-value**
*Age*	≤59 years	10	0.025	5	0.017
	60 or older	3		2	
*RPA class*	2	10	0.003	5	0.043
	3	3		2	
*Surgery*	Yes	16	0.049	10	0.091
	No	5		2	
*Therapy sequence*	IPI after RT	11	0.015	6	0.019
	IPI before RT	3		2	
**Multivariate analysis**					
		**Overall survival** **Hazard ratio**	***p*** **-value**	**Cerebral progression-free survival** **Hazard ratio**	***p*** **-value**
*RPA class*	2	–	0.003	–	0.13
	3	3.3		(1.76)	
*Therapy sequence*	IPI after RT	–	0.01	–	0.045
	IPI before RT	2.7		2.1	

*RPA* recursive partitioning analysis, *IPI* ipilimumab, *RT* radiotherapy

The side effect profile of patients receiving the combination of IPI and radiotherapy appeared not to be systematically different from that of patients receiving brain radiotherapy alone (STX, WBRT or both), as far as this was assessable given the limited availability of toxicity data. As maximum acute reactions, mild symptoms of increased intracranial pressure were observed. These were never life-threatening (i. e., grade 4). One patient developed radiation necrosis, which resolved spontaneously. This patient is one of the long-term survivors and at the time of writing was still alive.

## Discussion

The most remarkable finding of our study is the long-term survival (i. e., at least 36 months) of 4 patients after combined radiotherapy and IPI for brain metastases. Furthermore, our data is strongly suggestive of the superiority of a therapy sequence of initial radiotherapy followed by IPI, a finding which is at variance with some experimental data (see [9] for a comprehensive review). Our finding might have an impact on both the design of further clinical studies and for the routine use of IPI in conjunction with radiotherapy. Further studies need to evaluate whether the sequence of radio- and immunotherapy also applies to PD1/PD-L1 inhibition.

Two phase III trials demonstrated that IPI significantly improved OS both in previously treated and untreated patients with malignant melanoma [10, 11]. However, both trials excluded patients with active brain metastases. The study by Hodi and colleagues [10] allowed the inclusion of patients with previously treated and locally controlled melanoma brain metastases (MBM).

The efficacy of IPI specifically towards brain metastases was investigated in a prospective phase II trial by Margolin et al. [12]. Two groups of patients were analyzed. Group A (*n* = 51) consisted of asymptomatic patients, while group B (*n* = 21) comprised patients with neurological symptoms which could be controlled by corticosteroid treatment [12]. Approximately half of the patient also received radiotherapy. Disease control in the brain was achieved in 24% of patients in group A and 10% in group B. However, the effect of radiotherapy on this primary endpoint of the study was not specifically analyzed. Margolin et al. [12] concluded that one possible reason for the poor outcome of group B might have been a negative effect of corticosteroid treatment on IPI activity. However, even in corticosteroid naïve patients, IPI monotherapy does not always show a major benefit on the OS of patients with MBM as demonstrated in a retrospective study of 38 patients with asymptomatic MBM taking part in the French expanded access program [13]. In this latter study, the median survival was 101 days and 1‑year survival did not exceed 10.5%. The analysis revealed only a partial remission in 3 of 38 patients. Only 10 of the 38 patients in this report received radiotherapy. The authors concluded that the missing effect of IPI in the study population might be due to underdosing of IPI (3 mg instead of 10 mg/kg) in the heavily pretreated patient population presenting mainly in RPA (recursive partitioning analysis) class 2. However, another study using IPI at 3 mg/kg has reported positive results (see below) and our patient population also exclusively consisted of RPA class 2 and 3 (Table 1).

Data from prospective trials explicitly investigating the role of different combinations of IPI and radiotherapy for MBM are still pending (NCT01950195, NCT02662725, NCT01703507, NCT02097732, see https://clinicaltrials.gov). Therefore, information regarding this particular question can currently only be derived from retrospective analyses. The first report on this topic was published by Knisely et al. [14], who analyzed the records of 77 patients treated with gamma knife stereotactic radiosurgery for MBM at Yale-New Haven Hospital between 2002 and 2010. Of these patients, 27 also received IPI at some point during their treatment and showed a median survival of an impressive 21.3 months, while those who did not had a median survival of only 4.9 months (*p* = 0.044). At this institution, IPI was administered to patients in clinical trials since 2004. Knisely et al. [14] found no differences in the outcome of patients who had received IPI before or after radiotherapy. The topic was further investigated by Silk et al. [15]. Of 70 patients who received whole brain radiation therapy (WBRT) or stereotactic radiosurgery (SRS) between 2005 and 2012, 33 also received IPI. The latter patients had a median survival of 18.3 months compared to 5.3 months in the 37 patients who did not receive the drug. Similar to our findings, OS was substantially longer when IPI was given after vs. before radiotherapy (18.4 months vs. 8.1 months). However, contrary to our data, a significantly better outcome was noted for patients treated with SRS compared with WBRT. The impact of IPI on survival was independent from other prognostic factors in both studies, albeit only when censoring data at 24 months in the work of Knisely et al. [14]. In contrast to the findings above, Mathew et al. [16] retrospectively investigated a cohort of 58 patients treated with SRS, 25 of which received IPI, and found no difference in intracerebral disease control between patients receiving or not receiving IPI.

Additional studies have explicitly investigated the relevance of the sequence of IPI and radiotherapy without comparing results with patients who received irradiation alone. To the best of our knowledge, Kiess et al. [17] reported the largest series of these. Consistent with our findings, these authors showed that OS was better for patients who had received IPI during or after SRS compared with those who received IPI before SRS. Importantly, contrary to the present work, none of these studies investigated a cohort of patients who received IPI within a very short period of time before or after radiotherapy (i. e., median of 2 months in our cohort)—critical for a possible pathophysiological interaction between the two treatment modalities.

As reviewed by Patel et al. [18] and Demaria et al. [19], radiotherapy can stimulate immune responses via a number of pathways, e. g., enhanced antigen presentation or the release of danger-associated molecular patterns (“DAMPs”). It seems mechanistically plausible that this initial induction or boost of the antitumoral immune response may be disinhibited by subsequent immune checkpoint inhibition. Conversely, the opposite sequence of initial immune stimulation in the context of a low basal antitumoral activity of the immune system, followed by radiotherapy to the brain, which often requires corticosteroid administration, may be counterproductive. Given the fact, however, that we have no original data proving or disproving either possibility, these considerations are currently purely speculative.

In summary, we found that the sequence of radiotherapy followed by IPI was superior to IPI followed by radiotherapy for the treatment of brain metastases. We have identified long-term survivors after this combined treatment, with no neurological sequelae and a good quality of life. We did not observe a difference between SRS with high single doses compared to WBRT with moderate hypofractionation, although our patient number is still rather low. Our observations indicate that radiotherapy with moderate hypofractionation might already condition patients for anti-CTLA-4 therapy. Although statistically not significant, there was a remarkable difference in pretreatment with BRAF inhibitors between the two therapy sequence groups. The possibility cannot be ruled out that this might have influenced our findings concerning survival differences. Our retrospective analysis supported the hypothesis that sequence of radiotherapy and IPI might influence therapeutic outcome. Further retrospective studies should be evaluating treatment sequence as a possible prognostic factor in the combined treatment with radiation and checkpoint inhibitors. If our findings should be confirmed, prospective studies should evaluate the optimal time interval between the two treatments.
